# Patterns of Recent National Institutes of Health (NIH) Funding in General Surgery: Analysis Using the NIH RePORTER System

**DOI:** 10.7759/cureus.4938

**Published:** 2019-06-19

**Authors:** Erich J Berg, John Ashurst

**Affiliations:** 1 Medicine, Arizona College of Osteopathic Medicine, Phoenix, USA; 2 Emergency Medicine, Kingman Regional Medical Center, Kingman, USA

**Keywords:** grant funding, general surgery, r01, national institutes of health (nih)

## Abstract

Introduction: The National Institutes of Health (NIH) is the world’s largest funding source for research, and the R01 grant is seen as a stepping stone to future granting opportunities for the physician-scientist. Recently, both a gender and degree disparity in scholarly activity has been highlighted in the medical literature.

Objective: To assess NIH R01 funding trends in general surgery over the last decade.

Methods: A retrospective review of general surgery funding was extracted from the NIH’s Research Portfolio Online Reporting Tools Expenditures and Results database from 2008 through 2017. All principal investigators (PIs) were categorized by gender and academic degree(s). Linear regression analysis assessed NIH grant funding trends over time and comparisons of groups were completed with the t-test.

Results: From 2008 to 2017, the NIH awarded 600 R01 grants and $272,669,397 to PIs in general surgery. The majority of R01 grants were awarded to males (76.33%; p < 0.01) and those holding a Doctorate of Medicine (MD) degree (58.33%; p < 0.01). No Doctorate of Osteopathic Medicine (DO) had received an NIH R01 grant during the time studied. No statistically significant trend could be established for the number of R01 grants awarded over time (p = 0.33), grants awarded to males or females over time (p = 0.73 and p = 0.18), or for those holding an MD or other type of degree over time (p = 0.30 and p = 0.39). Also, no statistically significant trend was established for increased grant funding over time (P = 0.88) but females and those holding an other type of degree (Doctorate in Philosophy (PhD), Doctorate in Science (DSc), Master of Public Health (MPH), etc.) experienced an increase in the total dollar amount of funding over the time studied (p < 0.01 and p < 0.01).

Conclusion: For the years studied, a gender and degree disparity exists for those receiving an NIH R01 grant in general surgery. However, an increase in total grant funding has been seen for both females and non-physician scientists over from 2008 through 2017.

## Introduction

Research has been a driving force in the advancement of medicine, and the United States accounts for more than half of the world’s funding [1]. The National Institutes of Health (NIH) is the world’s largest funding source with the R01 grant being the oldest and most awarded grant to primary investigators (PIs) [2]. In 2014, the R01 grant accounted for 49% of all NIH extramural funding and 53% of the National Heart, Lung, and Blood Institute's (NHLBI) annual budget [2]. Furthermore, the R01 grant is seen as an early career milestone that the academician can use to promote further granting opportunities. 

Recent data has shown that there was also a 19% decrease in the total amount of NIH-awarded grants to general surgeons from 2003 through 2013 [3]. Furthermore, many surgical subspecialties have noted a gender disparity in those being awarded NIH grants [4]. Not only has a gender disparity been noted, but recently, a degree disparity has also come forward. A recent article noted that the award rate for NIH grants coming from allopathic medical schools was 21% higher than their osteopathic counterparts, and that 0.1% of all active grants come from an osteopathic organization [5]. The purpose of this study was to assess NIH R01 grant funding trends in general surgery over the last decade. 

## Materials and methods

Study design

Following the waiver of institutional review board approval, the NIH Research Portfolio Online Reporting Tools Expenditures and Results (RePORTER) search engine (http://projectreporter.nih.gov) was queried for “general surgery” as a departmental keyword among new R01 grants issued between the fiscal years of 2008 and 2017. Each primary investigator (PI) was then categorized as having a medical degree (DO or MD), an "other" type of degree (Doctorate in Philosophy (PhD), Doctorate in Science (DSc), Master of Public Health (MPH), etc.), or dual graduate degrees (a combination of a medical degree and non-medical degree). Secondarily, the total dollar amount of the awarded grant and the gender of the PI were recorded. This was accomplished by completing a web search for the author and accessing the author’s public biography displayed by their affiliated institution. 

Statistical analysis

Sixty RO1 grants out of the 600 reviewed for this study were used to provide a kappa analysis for inter-rater reliability. The total R01 grant amount was rounded to the nearest dollar. The authors (JA, EB) reviewed primary investigators, dollar amounts, degree designation, and gender from the 60 R01 grants to assure accuracy and no discrepancies were noted. Comparisons of the proportions of gender and PI degree(s) from each year were determined by using simple descriptive statistics. Comparisons for each group were completed by using the t-test, and linear regression was used to assess the temporal relationships between gender, degree obtained, number of awards per year, and dollar amounts awarded over time.

## Results

Over the decade studied, a total of 600 NIH R01 grants were awarded to general surgery and had a total worth of $272,669,397. Of the grants awarded, 76.33% (458/600) were awarded to males (p < 0.01) but there was no statistically significant difference in the total dollar amount awarded to each gender (p = 0.35). When the degree of the awardee was considered, 58.33% (350/600) held an MD degree (p < 0.01) and 37.43% (131/350) of those MDs held dual degrees. Of those MDs who held a dual degree, 82.44% (108/131) were male (p < 0.01). No DO received an NIH R01 grant in the general surgery category during the decade reviewed.

**Figure 1:**
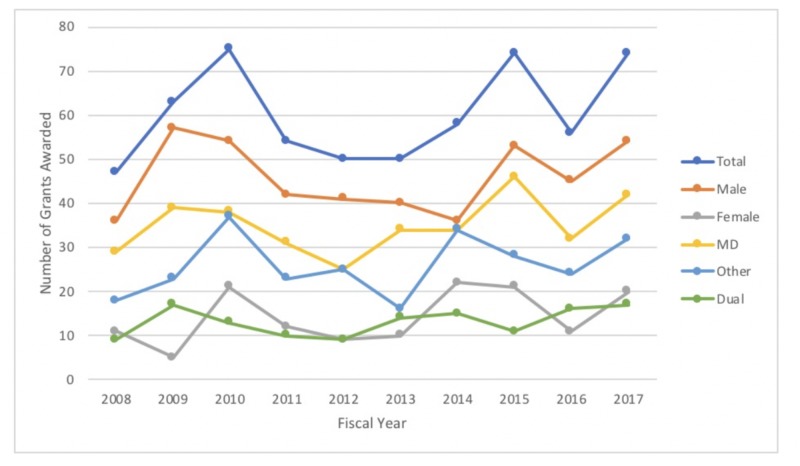
Number of NIH R01 grants awarded over the last decade in general surgery NIH: National Institutes of Health

No statistically significant trend could be established for the total number of grants awarded over the last decade (p = 0.33) (Figure 1). When gender was considered, no statistically significant trend was seen for either male or female grant recipients (p = 0.73 and p = 0.18, respectively). Furthermore, no statistically significant trend was noted when the type of degree of the recipient was considered (MD: P = 0.30 and Other: P = 0.39). 

**Figure 2:**
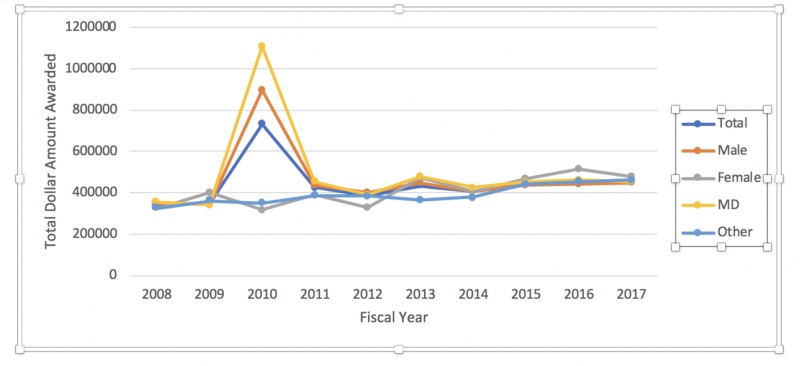
Dollar amount of NIH R01 grants awarded over the last decade NIH: National Institutes of Health

When the amount of award funds distributed annually was considered, no statistically significant trend could be established (P = 0.88). However, further subgroup analysis revealed a statistically significant positive trend for the female gender (p < 0.01) and those holding a degree listed as Other (P < 0.01) (Figure 2) for the years studied. No statistically significant trend was established for the male gender (P = 0.82) or those recipients holding an MD degree (P = 0.73) (Figure 2).

## Discussion

Much like emergency medicine, NIH R01 grant funding for general surgery has shown a degree disparity over the last decade [6]. The exact reasoning for this is unclear, but the lack of scholarly activity by osteopathic physician-scientists may play a key role in these findings. Currently, the NIH bio sketch requires perspective grant recipients to list “Contributions to Science” and “Scholastic Performance”. However, recent literature has shown that very few osteopathic physician-scientists publish manuscripts in high impact journals in the specialties of emergency medicine, obstetrics and gynecology, pediatrics, and neurosurgery and rarely serve on editorial boards of major journals [7-10]. Without these types of scholarly activity, it could be difficult for an osteopathic physician-scientist to meet the stringent NIH criteria. 

Further complicating matters, very few osteopathic physicians have served a role within the NIH. In 1997, 0.67% of all representation within the Department of Health and Human Services Federal Advisory Committees (DHHS FAC) were osteopathic physicians and a decrease in representation was then noted in 2017 to 0.27% [11]. When reviewing those who served on the Literature Selection Technical Review Committee in 2000, a significant degree disparity of committee members was also noted. Lastly, no osteopathic physician has ever served as either the Director or in the Director’s Office of the National Center for Complementary and Integrative Health (NCCIH) despite their purpose of funding grants which includes osteopathic terminology [11]. Without osteopathic physicians serving in these high-level positions at the NIH, it could be difficult for osteopathic physician-scientists to not only obtain an NIH grant but also be supplied with sufficient mentorship during the granting process. 

Not only was a degree disparity noted in our study but also a gender disparity. Females were less likely to receive an NIH R01 grant as compared to their male counterparts but have seen an overall increase in their total funding over the decade studied. Previous data in several subspecialties shows similar trends in which females have received significantly less NIH grants as compared to their male counterparts [4, 12]. However, no current studies in general surgery have shown an increase in funding for the female gender over the last decade. The exact mechanism is not clearly elucidated by this study but could be related to the original landmark study by Jagsi et al. showing a gender disparity in academic publications [13].

Being awarded an NIH R01 is a prestigious honor for the physician-scientist and the NIH receives a large number of applications yearly. The authors, however, cannot comment on the number of applications or rejections received by each gender or those with a specific medical degree that are not awarded by the NIH. The authors also relied on the NIH and each institution's website to gather information. If either of these were inaccurate, data may have been altered. The authors can also not comment on years or specialties that were not studied. Subspecialties within the surgical specialty were also not included in the final analysis and may have altered results if included. 

## Conclusions

Over the decade studied, a degree and gender disparity exist in the amount of total grants and award dollars that are being received by primary investigators for NIH R01 grants. However, there has been a trend for increased funding in those females who receive an NIH R01 grant. In order to combat these growing disparities, further research needs to be conducted to determine their cause. 
